# Supplementary material for: Hospital-Treated Infections and Increased Risk of Two EBV-Related Malignancies: A Nested Case-Control Study
Source: Cancers (Basel). 2022 Aug 5;14(15):3804. doi: 10.3390/cancers14153804 (PMC9367337; doi:10.3390/cancers14153804)
Supplement: Supplementary file 1 [file cancers-14-03804-s001.zip › cancers-1845973-supplementary.pdf]

Table S1: The Swedish revisions of International Classification of Diseases (ICD) codes for hospital-treated infections

	ICD-8	ICD-9	ICD-10
**Infection type**			
Bacterial	000, 001, 002, 003, 004, 005, 073, 076, 080, 081, 082, 083, 320, 362, 380, 381, 382, 383, 421, 461, 481, 482, 501, 510, 567, 590, 595, 597, 612, 613, 614, 616, 620, 622, 630, 635, 670, 678, 680, 681, 682, 684, 710, 720, 010, 011, 012, 013, 014, 015, 016, 017, 018, 019, 020, 021, 022, 023, 024, 025, 026, 027, 030, 031, 032, 033, 034, 035, 036, 037, 038, 039, 090, 091, 092, 093, 094, 095, 096, 097, 098, 099, 100, 101, 102, 103, 104, 390, 391, 392	001, 002, 003, 004, 005, 073, 076, 077, 078, 079, 080, 081, 082, 083, 320, 381, 382, 383, 383, 421, 461, 475, 481, 482, 510, 567, 590, 595, 597, 670, 730, 010, 011, 012, 013, 014, 015, 016, 017, 018, 020, 021, 022, 023, 024, 025, 026, 027, 030, 031, 032, 033, 034, 035, 036, 037, 038, 039, 040, 041, 090, 091, 092, 093, 094, 095, 096, 097, 098, 099, 100, 101, 102, 103, 104, 390, 391, 392, 614, 615, 616, 680, 681, 682, 683, 684, 685, 686	A00, A01, A02, A03, A04, A05, A15, A16, A17, A18, A19, A20, A21, A22, A23, A24, A25, A26, A27, A28, A30, A31, A32, A33, A34, A35, A36, A37, A38, A39, A40, A41, A42, A43, A44, A45, A46, A47, A48, A49, A50, A51, A52, A53, A54, A55, A56, A57, A58, A65, A66, A67, A68, A69, A70, A71, A72, A73, A74, A75, A76, A77, A78, A79, B95, B96, G00, G01, H60, H70, I00, I01, I02, I33, J01, J13, J14, J15, J36, J86, K65, L00, L01, L02, L03, L04, L05, L06, L07, L08, M00, M86, N30, N34, N70, N71, N72, N73, N74, N75, N76, N77, O23, O85, O86, P36
Viral	075, 360, 420, 422, 460, 464, 465, 466, 480, 040, 041, 042, 043, 044, 045, 046, 050, 051, 052, 053, 054, 055, 056, 057, 060, 061, 062, 063, 064, 065, 066, 067, 068, 070, 071, 072, 073, 074, 075, 076, 077, 078, 079, 470, 471, 472, 473, 474	070, 071, 072, 074, 075, 077, 078, 079, 372, 420, 422, 460, 464, 465, 466, 480, 487, 647, 711, 045, 046, 047, 048, 049, 050, 051, 052, 053, 054, 055, 056, 057, 060, 061, 062, 063, 064, 065, 066	A08, A60, A80, A81, A82, A83, A84, A85, A86, A87, A88, A89, A90, A91, A92, A93, A94, A95, A96, A97, A98, A99, B00, B01, B02, B03, B04, B05, B06, B07, B08, B09, B15, B16, B17, B18, B19, B20, B21, B22, B23, B24, B25, B26, B27, B28, B29, B30, B31, B32, B33, B34, B97, B99, H10, I30, I40, J00, J04, J05, J06, J10, J12, J20, J21, O98, P35, Z21
Others	084, 085, 086, 087, 088, 089, 112, 113, 114, 117, 120, 121, 122, 124, 125, 126, 128, 130, 131, 132, 133, 134, 135, 136, 363, 610, 611, 615, 732, 763, Y41	084, 085, 086, 087, 088, 112, 113, 114, 117, 118, 120, 121, 122, 124, 125, 126, 128, 130, 131, 132, 133, 134, 135, 136, 370, 675, 771, 137, 138, 139	V02, B37, B38, B45, B46, B47, B48, B49, B50, B51, B52, B53, B54, B55, B56, B57, B58, B60, B61, B62, B63, B64, B65, B66, B67, B68, B69, B70, B72, B73, B74, B75, B76, B77, B78, B79, B80, B83, B85, B86, B87 B88, B89, B90, B91, B92, B93, B94, H16, H32, M01, M02, M03, O91, P37, P38, P39, Z22, A59, A63, A64
**Infection site**			
CNS	013, 062, 063, 064, 065, 066, 071, 094, 292, 320, 323, 324, 390, 474, 040, 041, 042, 043, 044, 045, 046	013, 062, 063, 064, 071, 094, 320, 323, 326, 392, 045, 046, 047, 048, 049	A17, A80, A81, A82, A83, A84, A85, A86, A87, A88, A89, G00, G01, G02, G04, G05, I02
Gastrointestinal	014, 123, 127, 129, 540, 567, 000, 001, 002, 003, 004, 005, 006, 007, 008, 009	014, 123, 127, 129, 540, 567, 000, 001, 002, 003, 004, 005, 006, 007, 008, 009	A00, A01, A02, A03, A04, A05, A06, A07, A08, A09, B71, B81, B82, K35, K65, K67
Genitourinary	016, 590, 595, 597	016, 590, 595, 597	N30, N34, O23
Respiratory	010, 011, 012, 033, 034, 075, 115, 116, 490, 501, 503, 510, 460, 461, 462, 463, 464, 465, 466, 470, 471, 472, 473, 474, 480, 481, 482, 483, 484, 485, 486	010, 011, 012, 033, 034, 075, 115, 116, 473, 475, 487, 490, 510, 460, 461, 462, 463, 464, 465, 466, 480, 481, 482, 483, 484, 485, 486	A15, A16, A37, A38, B27, B39, B40, B41, B42, B44, B59, J00, J01, J02, J03, J04, J05, J06, J10, J12, J13, J14, J15, J16, J17, J18, J20, J21, J22, J32, J36, J40, J41, J42, J86, P23
Skin	110, 111, 050, 051, 052, 053, 054, 055, 056, 057, 680, 681, 682, 683, 684, 685, 686	110, 111, 050, 051, 052, 053, 054, 055, 056, 057, 680, 681, 682, 683, 684, 685, 686	B00, B01, B02, B03, B04, B05, B06, B07, B08, B09, B35, B36, B43, L00, L01, L02, L03, L04, L05, L06, L07, L08
